# The Role of Eating Behaviours in Genetic Susceptibility to Obesity

**DOI:** 10.1007/s13679-020-00402-0

**Published:** 2020-09-03

**Authors:** Moritz Herle, Andrea D. Smith, Alice Kininmonth, Clare Llewellyn

**Affiliations:** 1 Department of Biostatistics & Health Informatics, Institute of Psychiatry, Psychology & Neuroscience, Kings College London, 16 De Crespigny Park, London, SE5 8AF UK; 2 Research Department of Behavioural Science and Health, University College London, London, UK; 3 School of Psychology, University of Leeds, Leeds, UK

**Keywords:** CEBQ, Appetite, Appetitive traits, Eating behaviours, Genetics, Obesity, Twin studies, Polygenic risk scores, Behavioural susceptibility

## Abstract

**Purpose of Review:**

Eating behaviours are hypothesised to be the behavioural expression of genetic risk of obesity. In this review, we summarise findings from behavioural genetic research on the association between genetic risk for obesity and validated psychometrics measures of eating behaviours in children and adults (published in the past 10 years).

**Recent Findings:**

Twin studies have produced some evidence for a shared genetic aetiology underlying body mass index and eating behaviours. Studies using measured genetic susceptibility to obesity have suggested that increased genetic liability for obesity is associated with variation in obesogenic eating behaviours such as emotional and uncontrolled eating.

**Summary:**

More research on this topic is needed. Especially longitudinal studies using genetically sensitive designs to investigate the direction of genetic pathways between genetic liability of eating behaviours to weight and vice versa, as well as the potential subsequent link to eating disorders.

## Introduction

Obesity rates continue to rise globally and, to date, no country has yet been able to reverse this trend [1]. This is of concern given the considerable economic and social costs attributable to the increased morbidity and mortality associated with obesity. A comprehensive analysis of the direct healthcare costs and associated loss in economic activity estimated the total cost of obesity to amount to $2 trillion annually [2], which is roughly equivalent to 2.8% of the world’s gross domestic product. Current trends estimate that global obesity prevalence remains at around 13% of adults (aged > 18 years), but this increases to 39% of adults if the prevalence of overweight and obesity is combined [3]. In addition, childhood obesity is on the rise; with approximately 40 million children under 5 years of age being classified as having overweight or obesity in the year 2018. Evidently, the obesity pandemic is one of the biggest global public health challenges, and better understanding of the aetiology of obesity remains a key public health priority.

Changes to the food and activity environments such as increased availability of energy dense foods and more sedentary lifestyles have been implicated as key factors in driving the obesity epidemic [1, 4]. However, not everyone develops obesity, and, in fact, there is considerable variation in adiposity, indicating that there are large individual differences in susceptibility to the modern ‘obesogenic’ environment. Twin and family studies have shown that when individuals are well-matched for their environments, variation in human body weight—as well as rate of weight gain—has a strong genetic basis from infancy through adulthood [5–7]. Between 47 and 90% of inter-individual differences in weight are attributable to genetic differences between people [5]. These behavioural genetic studies provided insights into heritability patterns of body weight derived from twin and family studies, rather than molecular genetic studies that focus on mutations or smaller variations in or near specific genes.

Early functional and candidate gene studies attempted to identify major mutations in single genes that were implicated in the regulation of human body weight, or risk for severe obesity—so-called ‘monogenic’ forms of obesity. These candidate genes were largely found to code for key components involved in the central appetite regulation pathways (e.g. LEP; the gene coding for the satiety regulating hormone Leptin), and were rare in the population and observed only among individuals who developed severe, early onset obesity [8]. With technological advances, research increasingly focused on the identification of common genetic variants (in the form of single nucleotide polymorphisms, SNPs) using the genome-wide association study (GWAS) design. This hypothesis-free approach enabled the study of the architecture of complex common genetic variants and their contribution in the aggregate to polygenic obesity [9]. The first common genetic variant to be discovered, and the variant with the largest effect size to date, was a polymorphism in the *FTO* gene—which has been linked to weight, as well as behavioural and psychometric measures of satiety regulation [10]. Whilst *FTO* is believed to be a gene involved in individual predisposition towards the hyperphagic phenotype [11], there is also a growing amount of evidence that candidate studies in complex diseases are often underpowered and fail to account sufficiently for population stratification [12]. In addition, results from GWAS studies can be used to construct polygenic scores, which can be employed in epidemiological research as well as causal inference designs such as Mendelian randomisation [13]. As time went on, GWAS studies have indicated that variation across the whole genome is contributing to variation in human body weight, not just a few variants in easily identifiable genes. GWAS have identified ~ 100 genetic variants associated with BMI—and taken together these variants are able to explain ~ 6% of variance in BMI [9]. Considering most state-of-the-art full genome sequencing technology, the aggregate of all analysed genetic variances is much larger and can explain about 40% of BMI variance [14]. Genetic research into obesity has progressed substantially; however, genetic factors cannot explain the rapid increase of obesity rates across the globe. Therefore, there is a need for contemporary behavioural genetic research to understand the mechanism underlying genetic and environmental factors in shaping risk for obesity [15].

A key theoretical framework called the behavioural susceptibility theory (BST) hypothesises that genetic factors influence individual differences in weight through variation in appetite regulation, which is expressed as a range of distinct eating behaviours [16]. Central to the BST is the assumption that eating behaviours are key mediating mechanisms through which both genetic and the environmental factors influence variation in body weight. Obesity is proposed to result from a combination of genetic susceptibility to overeating and the environmental opportunity to overconsume, ultimately leading to positive energy balance. A steadily growing body of research has established that variation in eating behaviours emerges early is associated with individual differences in rate of weight gain and body weight, and that variation in eating behaviours is heritable from infancy onwards [17].

Researchers have been studying the relationship between eating behaviours and weight for nearly half a century leading to several well-established psychometric measures. The most common ones, and the focus of this review, are the Baby Eating Behaviour Questionnaire (BEBQ) [18], Child Eating Behaviour Questionnaire (CEBQ) [19], Dutch Eating Behaviour Questionnaire (DEBQ) [20], or Three Factor Eating Behaviour Questionnaire (TFEQ) [21]. All these psychometric tools were originally developed in white western European population but have been since adopted and validated for other populations. The BEBQ and CEBQ both consist of a number of subscales to capture eating behaviours in infancy and childhood. Their subscales can be broadly split into ‘food approach’ and ‘food avoidant’ eating behaviours. The food approach behaviours in the CEBQ are enjoyment of food, food responsiveness, emotional overeating, and desire to drink. Food avoidant behaviours are as follows: food fussiness, emotional undereating, slowness in eating, and satiety responsiveness. The BEBQ measures four of these scales: food responsiveness, satiety responsiveness, enjoyment of food, slowness of eating, and a single item measuring ‘general appetite’. In contrast, the DEBQ and TFEQ were originally developed for use in adults but have since been adapted for children and adolescents [22, 23]. Both measure three eating behaviours—the DEBQ measures external eating (eating in response to an external food stimuli), emotional eating (tendency to overeat in response to an emotional state), and restrained eating (deliberate suppression of eating), whereas the TFEQ measures dietary restraint, disinhibition, and hunger. These widely used psychometric measures made it possible to study eating behaviours at population level in children and adults has deepened our understanding of how eating behaviours may influence susceptibility to weight gain or loss.

The aim of this review is to synthesise the findings from behavioural genetic research over the past 10 years examining the links between eating behaviours and genetic susceptibility to obesity.

## Method

We conducted a scoping literature review in March 2020 of four databases (PsycINFO, EMBASE, Science Direct, and PubMed). Search terms were adapted for each database. All observational study designs (i.e. cross-sectional and prospective studies) were eligible for this review. Studies that focused on clinical samples were excluded. Only original research studies in English were included.

Reference lists and forward/backward citation tracking of included studies and relevant systematic review articles was also performed. Selected full-text articles were independently screened for inclusion by AS, AK, and MH using the following criteria: (1) the study needed to be a twin study or use a polygenic score, (2) the sample could include both child and/or adult populations, (3) eating behaviours needed to be measured using the BEBQ [18], CEBQ [19], DEBQ [20], or TFEQ [21] only in relation to an adiposity or weight-related outcome, and (4) studies had to be published in the last 10 years (2010–2020). Studies were *excluded* if they (1) were a candidate gene study, e.g. studies focusing on FTO, (2) were a family study (no including twin pairs), (3) focused on a clinical population (e.g. patients with eating disorders), or (4) investigated ‘clinical’ eating behaviours (i.e. disordered eating such as binge eating or eating disorders, e.g. the Eating Disorder Examination questionnaire [24])

Studies were subsequently divided according to their study design, and the age of the population studied (adults vs children). Narrative synthesis was undertaken to summarise the result of the review according to these categories. A list of all studies included can be found in Table 1.

**Table 1:** List of all studies included in the review

Study	Design	Country	Sample/cohort	*N*	Sex (% F)	Age	Eating behaviours	Measures	Interactions by sex	Genetic instrument
Keskitalo et al., 2008	Twin study	UK, Finland	UK Adult Twin Registry and FinnTwin12	314 MZ 327 DZ	83	17–82 years	Cognitive restraint, uncontrolled eating, emotional eating, food preferences, height, and weight	TFEQ, BMI	n/a	NA
Sung et al., 2012	Twin study	South Korea	The Healthy Twin study	443 MZ 124 DZ	60.3	20–65 years	Cognitive restraint, emotional eating, external eating	DEBQ, BMI	n/a	NA
Herle et al., 2020	Twin study	Spain	Murcia Twin Register	175 MZ 170 DZ	100	43–69 years	Cognitive restraint, uncontrolled eating, emotional eating	TFEQ, BMI	n/a	NA
Llewellyn et al., 2012	Twin study	UK	Gemini	4804	50.6	3 months	Enjoyment of food, satiety responsiveness, food responsiveness, slowness in eating	BEBQ, weight	No significant sex interaction	NA
Cornelis et al., 2013	Polygenic score	USA	The Nurses’ Health Study/The Health Professionals Follow-Up Study (HPFS)	3852	61.8	30–75 years	Cognitive restraint, uncontrolled eating, emotional eating	TFEQ, BMI	n/a	PGS-BMI, 32 SNPs
Konttinen et al., 2015	Polygenic score	Finland	DILGOM/FinnTwin12	5854	54	21–74 years	Uncontrolled eating, emotional eating	TFEQ, BMI	No significant sex interaction	PGS-BMI, 90 SNPs
Kontinnen et al., 2018	Polygenic score	Finland	DILGOM	3735	55	25–75 years	Uncontrolled eating, emotional eating	TFEQ, BMI	No significant sex interaction	PGS-BMI, 97 SNPs
de Lauzon-Guillain et al., 2018	Polygenic score	UK, France	Fenland, EDEN cohort	5669	Fenland: 53 EDEN: 55.7	Fenland: 51 years EDEN: 31 years	Cognitive restraint, uncontrolled eating, emotional eating	TFEQ, BMI	Sex modified the relationship between the BMI-GRS and cognitive restraint in Fenland (P-interaction = 0.02) but not uncontrolled eating (Fenland: *p* = 0.34; EDEN: *p* = 0.89) or emotional eating (Fenland: *p* = 0.26; EDEN: *p* = 0.57).	EDEN: PGS-BMI, 27 SNPs, 96 Loci in Fenland Fenland: PGS-BMI 96 SNPs
Jacob et al., 2018	Polygenic score	Canada	Quebec Family Study	768	43	43.3 years	Cognitive restraint, uncontrolled eating, emotional eating, dieting behaviour, avoidance of fattening foods, loss of control of eating in habitual, emotional and situational cues, susceptibility to hunger, and internal and external locus of hunger	TFEQ, BMI	No significant sex interaction	PGS-BMI, 97 SNPs
Llewellyn et al., 2014	Polygenic score	UK	Twins Early Development Study	2258	53.3	9.9 years	Satiety responsiveness	CEBQ, BMI	n/a	PGS-BMI, 28 SNPs
Steinsbekk et al., 2016	Polygenic score	Norway	Trondheim Early Secure Study	652	49.5	6 years	Enjoyment of food, satiety responsiveness, food responsiveness, slowness in eating, emotional overeating	CEBQ, BMI	n/a	PGS-BMI, 32 SNPs
Monnereau et al., 2017	Polygenic score	Netherlands	Generation R	3031	50	4 years	Food responsiveness, enjoyment of food, food fussiness, satiety responsiveness, and slowness in eating	CEBQ, BMI	n/a	Child obesity PGS-BMI, 15 SNPs Adult PGS-BMI, 97 SNPs
Abdulkadir et al., 2020	Polygenic score	UK	Avon longitudinal study of parents and children	4530	55.5	14 years	Emotional eating, external eating, restraint eating	DEBQ, BMI	No significant sex interaction	PGS-BMI derived with PRSice, see Ref [25]

### Twin Studies

The aim of twin studies is to estimate the relative importance of genetic and environmental influence on variation in any measured trait (e.g. body weight). The basis of the twin method is to compare the similarity of monozygotic twins (MZs), who share 100% of their genetic material, with dizygotic twins (DZs), who share on average 50% of their segregating genes. The twin method relies on the assumption that both types of twins share their environments to a similar extent (e.g. both have the same parents and grow up in the same household etc.); thus, any differences between MZs and DZs can only be attributed to the differences in their genetic relatedness. This approach allows for variation in a trait to be broken down into three latent factors. The proportion of variance in a trait that is explained by genetic variation is called ‘heritability’. Heritability ranges between 0% (no genetic role in trait variation) and 100% (variation in a trait is entirely explained by genetic factors). Heritability, or additive genetic effects, is commonly denoted with the letter A. In addition, the twin method allows us to separate the influence from the environment into shared environmental influences which includes all environmental factors contributing to increased similarity of two twins in a pair over and above genetic influences (e.g. the environment they grow up in at home) (denoted C), and non-shared environmental influences (denoted E) which include all environmental factors that contribute to within twin-pair differences, as well as measurement error (e.g. an illness that affects one twin only). Apart from estimating the relative contribution of genetic and environmental factors to *one* phenotype, the twin method can also provide information on the extent to which two phenotypes share their genetic and environmental aetiology. This is expressed as a genetic correlation (rA) which ranges from − 1 to + 1 and can be interpreted similarly to a Pearson’s correlation: e.g. if two phenotypes share all their underlying genetics and the genes that cause scores to be high on 1 trait also cause scores to be high on the other trait, rA = 1; if two phenotypes share all their underlying genetics but the genes that cause scores to be high on 1 trait cause scores to be low on the other trait, rA = − 1; if they share no genetic factors in common at all, rA = 0. Similarly, this method can also be used to investigate the effects of common environmental (rC and rE) influences; however, this is not the focus of this review. A more detailed description of the theory and statistical methodology underlying twin studies can be found elsewhere [26, 27]. For the purpose of this review, we have focussed on twin studies that examine the association between BMI (or any measure of adiposity) and eating behaviours, specifically on the reported genetic correlations between eating behaviours and BMI.

#### Adult Studies

One of the earliest studies, which used the twin method to examine the genetic correlations between eating behaviours and BMI, pooled data from two population-based cohorts; the UK adult Twin Registry (*n* = 1027; 17–82 years) and the Finnish twin research units (*n* = 299; 22–24 year) [28]. Eating behaviours were assessed using the TFEQ-R18, with cognitive restraint, uncontrolled eating, and emotional eating measured. Findings revealed significant genetic correlations between cognitive restraint and BMI (0.16), uncontrolled eating and BMI (0.29), and emotional eating and BMI (0.51).

These findings were supported in a study analysing data from the Healthy Twin cohort, a sample of same-sex twin pairs from South Korea. The sample consisted of 443 MZ and 124 DZ twin pairs, who were 20–65 years old [29]. Eating behaviours were assessed using a Korean version of the DEBQ. The results revealed significant genetic between objectively measured body weight (in kilograms) and restrained eating (0.31), emotional eating (0.32), and external eating (0.25). Together, these findings provide evidence for the genetic influence on associations between eating behaviours and weight in adults of European ancestry as well as those from East Asia.

However, a recent study of adults of European ancestry by Herle et al. (2020) [30] contradicted the findings from these previous two studies by Keskitalo et al. (2008) and Sung et al. (2012). This study analysed data from the Murcia Twin Registry, a population-based twin registry of same-sex female twin pairs (175 MZ, 170 DZ; aged 43–69 years) from southern Spain. Eating behaviours were measured using a Spanish version of the TFEQ-R18. There were no significant genetic correlations between eating behaviours and BMI, indicating no shared genetic aetiology between eating behaviours and adiposity in this sample. Rather, the relationship between eating behaviours and BMI appeared to be driven by non-shared environmental factors, with significant non-shared environmental correlations observed between BMI and both cognitive restraint (rE = 0.15) and uncontrolled eating and BMI (rE = 0.15). However, it is important to note that differences in findings between this study and the previous two studies may in part be due to geographical and socio-cultural differences between the study samples and age range (e.g. 17–82 vs 43–69 years). Furthermore, the lack of association may be due to low statistical power due to the relatively small sample sizes for twin-based estimates.

#### Child Studies

The only study in children to use the twin method to examine the extent to which associations between eating behaviours and weight are underpinned by shared genetic effects was conducted in a UK population-based cohort of infant twin pairs (724 MZ pairs, 1593 DZ pairs) born in 2007 [31]. Infants’ eating (or feeding) behaviours were assessed during the first 3 months of life in the period of exclusive milk-feeding, using the parent-reported BEBQ. Three feeding behaviours were included in the analysis: slowness in eating (SE), satiety responsiveness (SR), and a single item measuring overall appetite size (AS). Significant genetic correlations (rA) were observed between the three eating behaviours and weight at 3 months (0.22, 0.23, and 0.37 for SE, SR, and AS respectively). These results are in line with other twin studies which examined shared pathways between eating behaviours and adiposity in adults.

### Polygenic Score Studies

Twins studies decompose variation in an observable trait into genetic and environmental contributions, but do not involve studying specific measured genetic variants. With the advent of genomic sequencing, newer more advanced methods have been developed which enable cheaper and scaled-up research into the common genetic variants that contribute to differences in both BMI and eating behaviours. Polygenic scores (PGS) are aggregate scores which summarise genetic liability for a given phenotype. PGS are based on summary statistics of previous GWAS, which have been conducted to identify common genetic variants, specifically single nucleotide polymorphisms (SNPs), which are associated with a risk increase of illness or higher score on a quantitative measure [32, 33]. PGS are then derived by aggregating the weights estimated by GWAS, producing a risk score per individual from an independent sample. Importantly, this method allows researchers to investigate the extent to which common genetic variants associated with one phenotype are also linked to other phenotypes of interest. In comparison with twin studies, PGS studies indicate the degree to which an increase in the PGS is associated with a change in the outcome of interest, commonly reported as a regression beta coefficient. For the purpose of this review, we will focus on research which reported on the association between a change in PGS for BMI (PGS-BMI) and a change in eating behaviour scores. Some of the discussed studies expand their analyses, by including mediation and interaction analyses. When summarising these studies, these results are included as well. For clarification, mediation analysis intends to decompose the association between an exposure and an outcome (the total effect), into paths that go directly from exposure to outcome, versus paths that go via a third variable (the mediator). This association is commonly called the indirect effect. Doing so, mediation analysis aims to investigate a potential mechanism explaining why a total effect of exposure to outcome is observed. In contrast, interaction studies describe how a third variable exacerbates or attenuates an association between an exposure and an outcome. This is commonly done by stratifying the sample into discrete groups of interest and investigating if estimates differ between these [34].

#### Adult Studies

One of the first studies investigating the link between genetic liability for higher BMI with eating behaviours in adults combined data from two US cohorts: the Nurses’ Health Study (*n* = 2381; 30–55 years) and the Health Professionals Follow-Up Study (*n* = 1471; 40–75 years) [35]. Eating behaviours were assessed with the TFEQ and the genetic liability for obesity was indicated by adding up the carrier status of 32 common variants that had previously been associated with obesity. Significant associations were observed between genetic risk score and both higher emotional eating (*β* = 0.01; *p* < 0.01) and higher uncontrolled eating (*β* = 0.01; *p* < 0.01). There was no association between genetic liability for obesity and cognitive restraint (*β* = 0.006; *p* = 0.19).

These findings were replicated in a study pooling data from two population-based cohorts of men and women, Fenland (UK) and EDEN (Etude sur les déterminants pré et post natals précoces du Développement psychomoteur et de la santé de l’Enfant; France) cohort. The combined sample size was 5669 (Fenland: 53.2% women, EDEN: 55.7% women; age range = 18–64 years) and eating behaviours were measured with the TFEQ. PGS-BMI was based on 27 SNPs in EDEN and 96 SNPS in Fenland [36]. Results suggested that PGS-BMI was associated with higher emotional eating (EDEN *β* = 0.06, *p* = 0.01; Fenland: *β* = 0.04, *p* = 0.02) and uncontrolled eating (EDEN: *β* = 0.04, *p* = 0.04; Fenland: *β* = 0.06, *p* < 0.01). There was a positive association between BMI-PGS and cognitive restraint, but only for women (EDEN: *β* = 0.1, *p* < 0.01; Fenland: *β* = 0.07, *p* < 0.01). A mediation analysis revealed that uncontrolled eating and emotional eating partially mediated the association between PGS-BMI and BMI in both cohorts. After controlling for emotional eating, uncontrolled eating continued to independently mediate the association in Fenland cohort, but not EDEN. Further analyses revealed a significant interaction between BMI-PGS and cognitive restraint among men (EDEN; P-interaction < 0.001; Fenland *p* < 0.001) and among women in Fenland (*p* < 0.001) but not EDEN women. Neither emotional eating nor uncontrolled eating showed evidence of interaction with PGS-BMI. Overall, findings supported the hypothesis that common variants associated with higher BMI are also associated with variation in eating behaviours.

Similarly, a population-based study from Finland, which pooled data from two cohorts of men and women: the FinnTwin12 Study (*n* = 1231; 54% women; 21–26 years) and the DILGOM study (*n* = 4632; 53.8% women; 25–74 years) supported previous results showing that higher genetic risk for obesity (90 SNPs) was associated with higher emotional eating (*β* = 0.07; *p* < 0.001) and uncontrolled eating (*β* = 0.01; *p* < 0.001) as measured by the TFEQ [37], as well as with greater increases in restraint eating (*β* = 0.08, *p* < 0.001) in a 7-year follow-up study [38].

The most recent research using the TFEQ included 769 adults from the Quebec Family Study [39]. The PGS-BMI was calculated using 97 common variants and eating behaviours were assessed using a longer, more comprehensive version of the TFEQ, which divides cognitive restraint and disinhibition into several subscales, as well as measuring susceptibility to both internal and external hunger cues. Results proposed that higher PGS-BMI was associated with greater overall disinhibited eating (*β* = 0.14, *p* < 0.01), as well as susceptibility to both internal (*β* = 0.1, *p* = 0.02) and external hunger cues (*β* = 0.09, *p* = 0.03). In addition, authors investigated the extent to which eating behaviours mediate the association between PGS-BMI and weight outcomes. Results suggested that the association between PGS-BMI and BMI, as well as waist circumference, was partially mediated by all measured eating behaviours, supporting the notion that eating behaviours lie on the causal pathway from genetic liability to weight outcomes. However, due to the cross-sectional nature of the data, no causal inferences can be made.

#### Child Studies

The first study to use a polygenic score approach to investigate the association between genetic liability for obesity and eating behaviour in children analysed data from a population-based cohort, the Twins Early Development Study. The study included 2258 children, aged 10 years, who had data on eating behaviours measured with the CEBQ, as well as genotype information. The child-specific genetic liability for obesity was calculated from 28 SNPs identified in recent GWAS. Results highlighted how children with higher PGS-BMI had lower scores of satiety responsiveness (*β* = − 0.06, *p* < 0.05), indicating a higher appetite and lower receptiveness to internal feelings of fullness [40]. A mediation analysis indicated that weakened satiety responsiveness significantly mediated part of the association between the PGS-BMI and measures of adiposity (BMI and waist circumference).

Similarly, data from the Trondheim Early Secure Study, a longitudinal community-based cohort, was analysed to test the association between PGS-BMI based on 32 SNPs, and child eating behaviours measured by CEBQ, in 652 6-year-old children. Results indicated that a higher PGS-BMI was associated with faster eating rate. However, none of the other eating behaviours was associated with increased genetic liability for obesity at this young age [41]. It is also an unusually lean sample, and perhaps lower variation in BMI impacts statistical power in this already small sample. In addition, to these largely non-significant results, a following study analysed data from 3031 4-year-old children from the Generation R cohort in the Netherlands. This study aimed to find associations between PGS-BMI based on 15 SNPs from a previous childhood obesity GWAS, PGS-BMI based on 97 SNPs from previous adult obesity GWAS, and childhood eating behaviours measured with the CEBQ. Results found no associations between childhood-based PGS-BMI and eating behaviours. However, in line with Llewellyn et al. (2014), they observed a small but significant association between higher adult-based PGS-BMI and lower satiety responsiveness in children (*β* = − 0.007, *p* < 0.05). No other significant associations were reported [42]. Previous molecular genetic studies do not report an association between measured genetic risk of obesity and measures of adiposity in early childhood, so the younger age may explain the null or weaker findings [43].

One of the more recent studies analysed data from the Avon longitudinal study of parents and children, a longitudinal cohort study from the UK. Eating behaviours were measured using self-reported DEBQ when the participants were 14 years old (*N* = 4530) [44]. In comparison with previous research, this study used a more contemporary and comprehensive approach to derive the PGS-BMI. Instead of including only genome-wide significant SNPs from previous GWAS, the PGS-BMI was calculated with an iterative process including as many SNPs as possible. This is done by calculating several PGSs based on different *p* value thresholds, instead of just the genome-wide significant threshold of *p* = 10^−8^, such as *p* = 0.05, 0.01, and 0.1. The final polygenic score is chosen, because it explains the maximum amount of variance in the outcome [25]. Due to this method, the number of included SNPs is large and differs for each phenotype (emotional eating: 15,467 SNPs, restrained eating: 36,088 SNPs, and external eating: 10,780 SNPs). Results indicated that PGS-BMI was significantly associated with greater restrained eating (*β* = 0.14, *p* < 0.05), emotional eating (*β* = 0.21, *p* < 0.05), and lower external eating (*β* = − 0.19, *p* < 0.05). This negative association between the PGS-BMI and external eating at age 14 years seems counter intuitive at first. Further analyses highlighted that this association was mediated by BMI at 11 years, indicating that increased genetic liability for higher BMI was associated with greater BMI at 11 years which in turn was linked to lower external eating at 16 years. This result can be interpreted that participants with higher weight already at age 11 years change their eating behaviour aiming to reduce their external eating, e.g. the tendency to eat based on external food cues in the environment.

## Summary

In this review, we discussed a total of 4 twin studies and 9 studies using a polygenic instrument. In summary, these previous twin studies lend some support for BST as findings demonstrate significant common genetic influence on eating behaviour and weight. However, a lack of longitudinal twin research means the directionality of the relationship is not known. Furthermore, differences in findings between the adult twin studies highlight how estimates from twin research are sample specific, and how in some environmental and socio-economic contexts environmental factors might be dominant. In children, there has only been one twin study, which supported the BST, but this was in the earliest period of infancy. More research is needed in different samples and at other developmental stages. Furthermore, it is important to note that many of the studies in children rely on parent-report measures to observe child behaviours. This might be appropriate for young children, who are not yet able to self-report; however, more recently, child self-report measures of eating behaviours have been adapted and validated and should be considered for future research in this area [23, 45–47]. Overall, there has been a limited amount of studies investigating the association between genetic liability for obesity and eating behaviour in childhood. Furthermore, many of the conducted studies are small, with less than 1000 participants, and have focused on early childhood when associations between measured genetic susceptibility to obesity and adiposity are very small or not yet detectable. The two larger, more powered studies in older children have produced results suggesting that higher PGS-BMI is associated with lower responsiveness to internal satiety cues, supporting partially the behavioural susceptibility theory of obesity in children [40, 42]. One potential reason for the null findings in younger children might be that in early childhood, parents remain the main gatekeepers of children’s eating and might unconsciously, or purposefully, intervene. Hence, genetic propensity to overeating might still be suppressed by parental intervention.

For studies in adults, previous studies provide some support for the BST proposing that higher BMI-PGS is associated with greater food approach behaviour, such as emotional and uncontrolled eating. However, there was limited support that BMI-PGS is associated with measures of restraint or other dieting behaviour. Even though studies reported associations that met statistical significance cut-offs, reported effect sizes are small (*β* range: 0.001–0.09), with the greatest effect reported for the association between PGS-BMI and uncontrolled eating [37, 38]. As discussed in the above, PGSs are constructed from previous GWAS. In the case of BMI, the genome-wide significant SNPs can explain ~ 6% of the variance. Hence, the expected effect size for associations between PGS-BMI and other, even though correlated, behavioural phenotypes is expected to be small. This is different for more contemporary methods, as used by Abdulkadir et al. (2020), which substantially increase the explanatory power of the PGS studies. This approach resulted in higher estimates of the reported associations (*β* range = − 0.19, 0.21) and provides support for the BST, proposing that increase in genetic liability for obesity is associated with increases in eating behaviours such as emotional eating in adolescence [44]. Furthermore, this is the only study focussing on adolescence, which might be a crucial period in establishing independent health behaviours.

## Limitations

When reviewing the literature, the following limitations were identified. Overall, for both twin and PGS studies, sample sizes were relatively small. The power to estimate genetic correlations in twin studies is dependent on the heritability of the two phenotypes and the size of the true underlying genetic correlation. Simulations have demonstrated that in order to achieve > 80% power to detect a significant genetic correlation, with heritability estimates of 50% and an underlying genetic correlation of 0.3, a sample size of 500 is required. However, if the genetic correlation is lower, closer to 0.1, the sample size needs to be considerably larger, approximately > 3000 [48]. The sample size for some of the twin studies was small [28–30], leading to imprecise estimates with wide confidence intervals. In addition, previous research has mainly been based on white Western populations, and there was a dearth of research in this area in more diverse populations from different cultural contexts. This is crucial, as heritability estimates of eating behaviours and BMI are time and population specific. In other words, the heritability index is the indication of how much variation for a specific sample at a specific point in time is attributable to genetic factors. But it is well-established that heritability estimates change across the life course [49] which may explain some of the contradictory findings of the individual studies.

Similarly, sample size varied substantially between PGS studies (*N* range = 652–5854). Larger studies tended to combine samples from different longitudinal cohorts. Statistical genetics methods are constantly evolving, and procedures to derive polygenic scores from a few years ago might already be out-of-date. Crucially, PGS are based on previous GWAS, and hence if a new GWAS with a larger sample than the prior study is published, it is possible to calculate new, more comprehensive and therefore more powerful PGS. This dynamic nature of the method is reflected in the large variability on how PGS were derived in the studies included in this review. More recent methods are able to include all information from previous GWAS, instead of just focussing on the genome-wide significant SNPs, such as PRSice [25] or Linkage Disequilibrium Score Regression [50]. The potential of these methods is demonstrated by the findings from Abdulkadir et al. (2020), but replications in different populations are necessary [44]. In addition, more research is needed to investigate the extent to which PGS studies based on adult GWAS can be used in a childhood samples, as previous research has suggested that standard PGS-BMI were not associated with birth weight, and only weakly with weight in early life [43]. In addition, all PGS studies have been conducted in white population and there is an urgent need to expend this research into non-white, non-western populations.

In addition, the lack of longitudinal research currently hinders our understanding in the area. More longitudinal research using genetically sensitive designs are required to investigate the direction of genetic pathways between genetic liability of eating behaviours to weight or eating behaviours to weight and subsequently to risk to eating disorders.

## Conclusion and Future Directions

The reviewed literature gives some tentative support for the BST. However, small sample sizes, general lack of research in this area, lack of longitudinal research, and outdated methodology preclude a definitive conclusion. Future studies require larger sample sizes as well as the application of state-of-the-art statistical genetic methods to improve the statistical power. In addition, apart from estimates from twin research, so far, no GWAS has aimed to identify SNPs associated with eating behaviours directly. Similar behavioural phenotypes like this have been studied in this way with some success, such as food addiction and sugar intake [51]. In addition, previous research has linked eating behaviours in childhood to later eating disorders [52] and the association between emotional eating and binge eating disorder has been documented [53]. Hence, future research should expand and investigate the link between genetic liability for eating disorders, such as anorexia nervosa, and binge eating disorder.

Regardless of the genetic component to eating behaviours and obesity, environmental factors remain key intervention targets aiming to curb global obesity rates. Studies in this review indicate that there is a phenotypic, as well as potentially genetic, correlation between eating behaviours and BMI, suggesting that interventions aiming to change BMI might be successful when targeting eating behaviours.
